# Two controlled trials to increase participant retention in a randomized controlled trial of mobile phone-based smoking cessation support in the United Kingdom

**DOI:** 10.1177/1740774511416524

**Published:** 2011-10

**Authors:** Ettore Severi, Caroline Free, Rosemary Knight, Steven Robertson, Philip Edwards, Elizabeth Hoile

**Affiliations:** a Health Protection Agency, London, UK; b Nutrition and Public Health Intervention Research Unit, London School of Hygiene and Tropical Medicine, London, UK

## Abstract

***Background*** Loss to follow-up of trial participants represents a threat to research validity. To date, interventions designed to increase participants’ awareness of benefits to society of completing follow-up, and the impact of a telephone call from a senior female clinician and researcher requesting follow-up have not been evaluated robustly.

***Purpose*** Trial 1 aimed to evaluate the effect on trial follow-up of written information regarding the benefits of participation to society. Trial 2 aimed to evaluate the effect on trial follow-up of a telephone call from a senior female clinician and researcher.

***Methods*** Two single-blind randomized controlled trials were nested within a larger trial, Txt2stop. In Trial 1, participants were allocated using minimization to receive a refrigerator magnet and a text message emphasizing the benefits to society of completing follow-up, or to a control group receiving a simple reminder regarding follow-up. In Trial 2, participants were randomly allocated to receive a telephone call from a senior female clinician and researcher, or to a control group receiving standard Txt2stop follow-up procedures.

***Results*** Trial 1: 33.5% (327 of 976) of the intervention group and 33.8% (329 of 974) of the control group returned the questionnaire within 26 weeks of randomization, risk ratio (RR) 0.99; 95% confidence interval (CI) 0.88–1.12. In all, 83.3% (813 of 976) of the intervention group and 82.2% (801 of/974) of the control group sent back the questionnaire within 30 weeks of randomization, RR 1.01; 95% CI 0.97, 1.05. Trial 2: 31% (20 of 65) of the intervention group and 32% (20 of 62) of the control group completed trial follow-up, RR 0.93; 95%CI 0.44, 1.98.

***Conclusions*** In presence of other methods to increase follow-up neither experimental method (refrigerator magnet and text message emphasizing participation's benefits to society nor a telephone call from study's principal investigator) increased participant follow-up in the Txt2stop trial.

## Introduction

Randomized controlled trials (RCTs) generally are considered the gold standard in evaluation of health care interventions but losses to follow-up represent an important threat to both their internal and external validity [1–3]. Bias may occur as those lost to follow-up are likely to differ from those for whom follow-up is completed and bias may be more pronounced where there is differential follow-up between the intervention and control groups [4]. Sprague *et al*. [4] suggest that under 5% loss to follow-up will result in little bias, while a loss greater than 20% may represent a serious threat to the validity of the study. However, as the amount of bias introduced depends on the differences between those who were and were not followed-up and is trial specific, the level of bias caused by losses to follow-up cannot be accurately determined [4]. The potential for bias introduced by losses to follow-up can only be completely eliminated by achieving 100% follow-up, so it is imperative that trials aim to achieve as close to complete follow-up as possible [5].

Many trials fail to achieve high follow-up, potentially wasting economic and intellectual resources. A review of participant recruitment and retention in RCTs in six major journals from 2009 showed that 48% of trials reporting a sample size calculation failed to achieve adequate numbers at outcome assessment, once those lost to follow-up were excluded [6]. Economic and intellectual resources allocated to research studies are not limitless, and study validity compromised by excessive loss to follow-up is a waste of valuable resources.

A wide range of methods to increase response rates for postal, e-mail, and telephone questionnaires have been evaluated in RCTs, including the length of questionnaire, prenotification, number of requests, the nature and style of questions, incentives, status of sender, and method of delivery [7].

The effects on follow-up of interventions that emphasize trial participation's benefits to society are uncertain [7]. In all, 10 trials (with a total of 12,731 participants) evaluated the effect of an appeal stressing the benefit to society if participants return a questionnaire: pooled odds ratio (OR) 1.09 (95% confidence interval [CI] 0.92 to 1.29) [7]. The trials, however, are affected by several design weaknesses: none of the 10 trials gave evidence of the methods of randomization, except one [8], where allocation was not through randomization but systematic; there was no evidence that allocation of study arm was concealed from trial staff; and all the trials showed evidence of selection bias [7].

The effect of a telephone reminder from a senior female clinician/researcher on response rates is not clear. Trials sometimes use a senior staff member to contact non-respondents with the aim of reducing loss to follow-up. Completed trials showed no evidence of effect, when a senior or well-known person signed letters accompanying questionnaires: pooled ORs 1.05 (95% CI 0.89 to 1.23) and OR 1.05 (95% CI 0.95 to 1.15), respectively [7]. There was also no evidence of effect on response for the gender of the person requesting follow-up questionnaires (OR 1.07; 95% CI 0.72 to 1.58) but in one trial the odds of response decreased by over a half, when the electronic questionnaire was signed by a man (OR 0.55; 95% CI 0.38 to 0.80) [7]. It is plausible that personal contact by telephone may influence response rates, especially in a clinical trial where the call comes from a senior female clinician and researcher, but to date there have been no trials evaluating this approach.

Txt2stop is a RCT with 5800 participants that has been designed to evaluate the effect of mobile phone-based smoking cessation support on smoking rates at 6 months after enrolment. The eligibility criteria for Txt2stop were: aged 16 years or over; daily smoker; willing to quit in the next month; owned a mobile phone and resident in the United Kingdom. The primary outcome of Txt2stop was biochemically verified continuous abstinence at 6 months. Data regarding self-reported smoking status were collected and, from those reporting continuous abstinence, a salivary sample for cotinine level assessment was requested by post. Previous trials of smoking cessation support, particularly those using new technologies to deliver support, have experienced high and differential losses to follow-up for long-term outcomes [9,10]. One of our aims in the Txt2stop trial was to minimize loss to follow–up. We followed-up participants by any of the means they agreed to at the start of the trial, including post, e-mail, and telephone calls to mobile, home, or work numbers [11]. We used all the effective evidence-based methods that were feasible to introduce into the procedures of the trial [12], as identified in the systematic reviews by Edwards *et al*. and Hoile *et al*. [12,13]. These included monetary incentives, posting correspondence by recorded delivery, pre-notification, follow-up contact, unconditional advance cash incentives, short, concise questionnaires, duplicate questionnaires sent at repeat follow-up attempts, mentioning that commitment to the trial implied an obligation to respond, mention of university sponsorship, prepaid return envelopes with stamps, an assurance of confidentiality, and first-class outward mailing.

In addition to these procedures, we generated two hypotheses to test in two trials conducted among Txt2stop participants.

The first research hypothesis was that telling study participants that their participation in research could benefit society, would reduce loss to follow-up in the Txt2stop trial.

The second research hypothesis was that a telephone call from the study principal investigator (female senior clinician and researcher) explaining the importance of follow-up and asking participants to complete their participation would reduce loss to follow-up in the Txt2stop trial.

## Methods

We obtained ethical approval for these trials from the London School of Hygiene and Tropical Medicine Ethics Committee.

Study design and characteristics of the two trials are described in Table 1.

**Table 1:** Studies design and characteristics

	Trial 1	Trial 2
Study design	Single-blind randomized control trial	Single-blind randomized control trial
Intervention	Refrigerator magnet by post and telephone text message emphasizing social benefits of study participation	Telephone call from senior female clinician and researcher inviting participant to complete follow-up
Control	Text message reminding participant follow-up	Standard Txt2stop procedures
Eligibility criteria	Txt2stop participants enrolled between March 1 and June 1, 2009	Txt2stop participants >6 weeks overdue for cotinine sample follow-up
Trial consent	Consent was implicit in Txt2stop by choosing to provide follow-up or not	Consent was implicit in Txt2stop by choosing to provide follow-up or not
Withdraw	Available at any time withdrawing from Txt2stop	Available at any time withdrawing from Txt2stop
Allocation method	Minimization	Randomization
Primary outcome	Completed follow up at 30 weeks from randomization	Completed cotinine sample follow-up for the Txt2stop trial
Secondary outcome	Completed follow up at 26 weeks from randomization	No secondary outcome
Statistical analysis	RR for response and 95% CI. Test of homogeneity to evaluate a potential effect modification of being allocated to Txt2stop intervention or control group	RR for response and 95% CI
Sample size	1900 participants to detect a difference in follow-up at 30 weeks of 85% vs 80%; 80% power and 0.05 significance level (two-sided)	127 participants to detect a difference in follow-up at 35 weeks of 55% vs 30%; 80% power and 0.05 significance level (two-sided)

RR: risk ratio, CI: confidence interval.

*Trial 1* was a RCT evaluating the impact of an intervention, that is, providing information regarding the benefits to society of participation, on participants’ follow-up. This was a single-blind controlled trial, with those recording and assessing outcomes blind to the intervention.

**Figure 1:**
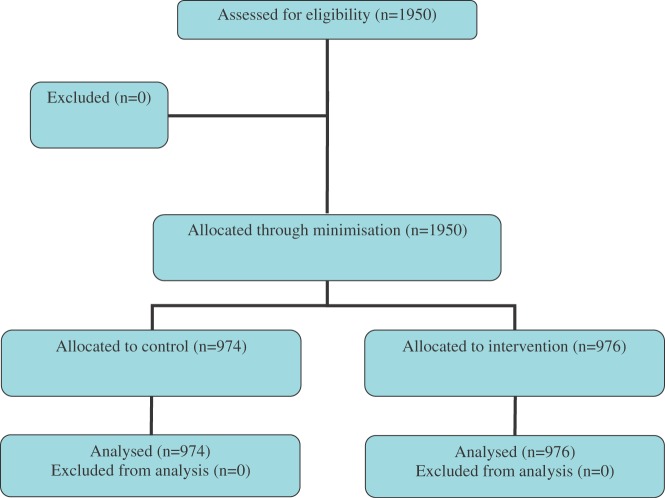
Trial 1 flow chart (a refrigerator magnet and a text message to increase participant's retention in Txt2stop)

In addition to the standard Txt2stop follow-up procedures, the intervention group was sent written information on a refrigerator magnet by post (see Figure 1), between 16 and 20 weeks after randomization into the Txt2stop trial, followed by a mobile phone text message 3 days after the Txt2stop postal follow-up questionnaire was sent. We aimed to sensitize participants by making them aware of the social benefits of remaining in the study for its full duration, regardless of smoking cessation status.

The initial sensitization consisted of a message on a refrigerator magnet within a sealed envelope. The message said that medical research is important to society and pointed out that by taking part in Txt2stop, the participant was benefiting society.

The text message said ‘Be proud of yourself for helping medical research! Thank you for filling in the Txt2stop questionnaire.’ The control group received a text message reminding the participant the follow-up questionnaire was due 3 days after the Txt2stop postal questionnaire had been sent (Week 23). The text message said ‘Thank you for filling in the Txt2stop questionnaire.’

The 1950 Txt2stop participants who enrolled between 1 March 2009 and 1 June 2009 and had provided postal addresses were eligible for Trial 1 [12]. They were able to withdraw at any time by texting ‘stop’ to the short code 65151. Any participant withdrawing from the Txt2stop trial was also withdrawn from Trial 1.

The participants were allocated to Trial 1 intervention or control through minimization (using Minim software [14] to balance four different characteristics affecting participant retention in Txt2stop: age, sex, Fagerstrom index (measure of nicotine dependency), and allocation to intervention or control group in Txt2stop. The allocation of the participants to Trial 1 intervention or control group was concealed from the investigators.

The primary outcome of the study was completed follow-up questionnaires at 30 weeks from randomization. The secondary outcome was completed follow-up questionnaires at 26 weeks from randomization.

The statistical analysis compared the proportions of those not lost to Txt2stop follow-up in intervention and control group using chi-square tests. A risk ratio (RR) for response and a 95% CI were calculated. A test of homogeneity also was used to evaluate potential effect modification of being allocated to Txt2stop intervention or control group.

The study was powered for the primary outcome measure. For a difference in follow-up at 30 weeks of 85% versus 80%, there was an 80% chance (power) that a trial with 1900 subjects, 950 per group, would detect the difference with two-sided probability ≤0.05.

*Trial 2* was a RCT of one telephone call from the study principal investigator to increase participant follow-up. Trial 2 also was a single-blind controlled trial, with those recording and assessing outcomes blind to the intervention.

**Figure 2 fig2-1740774511416524:**
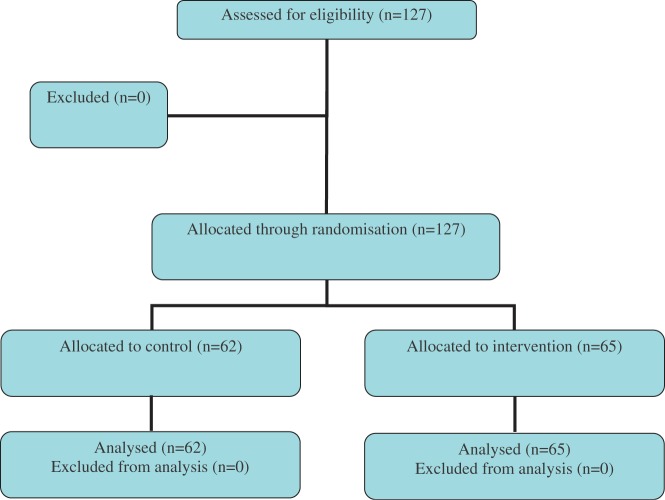
Trial 2 flow chart (a telephone call from the study principal investigator requesting follow-up in Txt2stop)

In addition to the standard Txt2stop procedures, the intervention group received a telephone call from the study principal investigator (female senior clinician and researcher), who invited participants who were at least 6 weeks overdue in providing a cotinine sample to complete follow-up. The control group received the standard Txt2stop procedures (see Figure 2).

All Txt2stop participants more than 6 weeks overdue for cotinine sample follow-up in April 2009 were eligible for Trial 2 [12]. As in Trial 1, participants had consented to join the Txt2stop trial and any participant withdrawing from the Txt2stop trial was also withdrawn from Trial 2. Allocation to Trial 2 intervention or control group was performed through computer-generated randomization and was concealed from the investigators. The study principal investigator contacted participants by telephone in April 2009.

The primary outcome of the study was completed cotinine sample follow-up at the end of May 2009 for the Txt2stop trial.

The statistical analysis compared the proportions of those not lost to follow-up in intervention and control group using chi-square tests. A RR for response and a 95% CI were calculated.

The study was powered for the primary outcome measure. For a difference in follow-up of 55% versus 30%, there was an 80% chance (power) that a trial with 127 subjects, 65 in intervention and 62 in control group, would detect the difference with two-sided probability ≤0.05.

## Results

*Trial 1*: 1950 participants were included in the trial. As shown in Table 2, the baseline characteristics of the Trial 1 population were similar to those of the Txt2stop population. Twenty six weeks after randomization, 33.5% (327 of 976) of participants in the intervention group posted the questionnaire back, compared with 33.8% (329 of 974) of participants in the control group. The RR for response to the refrigerator magnet was 0.99 (95% CI 0.88 to 1.12). The effect modification by allocation arm within the Txt2stop intervention and control groups was tested; the test of homogeneity showed no interaction (*p*-value = 0.92).

**Table 2:** Demographic characteristics of Txt2stop, Trial 1 and Trial 2 populations

Variable	Txt2stop population *N* (%)	Trial 1 population *N* (%)	Trial 2 population *N* (%)
Gender			
Women	2605 (44.9)	883 (45.3)	60 (47.2)
Men	3195 (55.1)	1067 (54.7)	67 (52.8)
Age (years)			
<30	1572 (27.1)	573 (29.4)	64 (50.4)
30–45	2716 (46.8)	897 (46.0)	50 (39.4)
>45	1512 (26.1)	480 (24.6)	13 (10.2)
Ethnicity			
White	5136 (88.5)	1771 (90.8)	109 (85.8)
Black	240 (4.1)	62 (3.2)	6 (4.7)
Asian	253 (4.4)	61 (3.1)	8 (6.3)
Other	134 (2.3)	45 (2.3)	3 (2.4)
Unknown	37 (0.6)	11 (0.6)	1 (0.8)
Age left school			
≤16	2538 (43.8)	985 (50.5)	54 (42.5)
>16	3262 (56.2)	965 (49.5)	73 (57.5)
Employment			
Manual	1789 (30.8)	523 (26.8)	28 (22.0)
Nonmanual	2539 (43.8)	679 (34.8)	45 (35.4)
N/A unknown	1472 (25.4)	748 (38.4)	54 (42.5)
Fagerstrom index			
≤5	3488 (60.1)	1154 (59.2)	59 (46.5)
>5	2312 (39.9)	796 (40.8)	68 (53.5)

After 30 weeks of randomization, 83.3% (813 of 976) of participants in the intervention group posted the questionnaire back, compared with 82.2% (801 of 974) of participants in the control group. The RR for response was 1.01 (95% CI 0.97 to 1.05). Again, no effect modification by allocation arm within the Txt2stop trial was detected (*p*-value from the test of homogeneity = 0.83).

*Trial 2*: A total of 127 participants were included in the analysis. As shown in Table 2, the baseline characteristics of the population in Trial 2 were similar to those of the population in Txt2stop in regard to sex and ethnicity. The Trial 2 population was younger and slightly more educated than the total Txt2stop population; a lower proportion were manual workers and fewer Trial 2 participants had serious nicotine dependence.

In all, 31% (20 of 65) of participants in the intervention group completed follow-up by sending cotinine samples, compared with 32% (20 of 62) in the control group. The RR was 0.93 (95% CI 0.44 to 1.98).

## Discussion

Information emphasizing the benefits to society, via written information on a refrigerator magnet and a subsequent text message, did not increase the return of completed questionnaires at 26 weeks or 30 weeks after randomization. A single telephone call from a female senior clinician and researcher did not increase return of mailed saliva samples. These interventions had no additional effect on follow-up when evaluated within a trial, where all other interventions known to increase follow-up [5,10] already had been implemented. These trials were pragmatic and, apart from the interventions tested, participants received all interventions to increase follow-up that were part of the Txt2stop standard operating procedures.

The impact of the intervention emphasizing the benefits to society of completing follow-up may have been reduced, since follow-up letters in the standard Txt2stop follow-up procedures already emphasized these benefits to some extent. The Txt2stop standard procedures included telephone calls from trial assistants, who could be male or female. The senior female clinician and researcher was not the only female member of staff calling Txt2stop participants.

For Trial 2, we included the entire sample of participants available at the time. With this sample, Trial 2 had sufficient power to detect an absolute difference of 25% between intervention and control groups. Ideally, trials should be designed todetect ‘meaningful’ differences in the outcome. Even modest increases in follow-up could reducebias. Depending on the existing level of trial follow-up, the number of trial participants, and the alternate priorities for a senior investigator's time, it could be worthwhile for a senior investigator to put in the time to increase follow-up by less than 25% (for example, a 5–10% absolute increase in follow-up could be important). A limitation of Trial 2 was that the trial was underpowered to detect an absolute increase in follow-up of less than 25%.

The Trial 1 control group received a text message very similar to the text sent to the Trial 1 intervention group but without any comment emphasizing benefits to society. This text message was sent because telephone text messages have been shown to work as effective reminders for appointments and medications reminders [15,16]. Several days of strike of the Royal Mail service during 3 weeks in October and November 2009 [17] seriously affected the return of Txt2stop questionnaire. The strike is likely to have decreased the return of questionnaires in the early phase. During the strike, it is possible that some returned questionnaires may have been lost by Royal Mail and attempts to obtain the data for a second time may have been less effective than the first request. There is no reason to expect that the strike would have differentially affected follow-up between the intervention and control group, and so the strike may have affected the precision of the result but is unlikely to have influenced the direction of effect.

We found no evidence that either emphasizing the benefits to society of trial participation to participants or a call from a female senior clinician and researcher influence follow-up, when other evidence-based methods to decrease loss to follow-up are already employed.

## Funding

This research received no specific grant from any funding agency in the public, commercial, or not-for-profit sectors.[Fig fig2-1740774511416524]

## References

[1] Akl EA Briel M You JJ LOST to follow up information in trials (LOST-IT): a protocol on the potential impact. Trials 2009; 10: 40–40 19519891 10.1186/1745-6215-10-40 PMC2706244

[2] Hunt JR White E Retaining and tracking cohort study members. Epidemiol Rev 1998; 20: 57–70 9762509 10.1093/oxfordjournals.epirev.a017972

[3] Villacorta V Kegeles S Galea J Innovative approaches to cohort retention in a community-based HIV/STI prevention trial for socially marginalized Peruvian young adults. Clin Trials 2007; 4: 32–41 17327244 10.1177/1740774506075869 PMC2853960

[4] Sprague S Leece P Bhandari M Limiting loss to follow up in a multicenter randomized trial in orthopedic surgery. Control Clin Trials 2003; 24: 719–25 14662277 10.1016/j.cct.2003.08.012

[5] Schulz KF Grimes DA Sample size slippages in randomised trials: exclusions and the lost and wayward. Lancet 2002; 359: 781–5 11888606 10.1016/S0140-6736(02)07882-0

[6] Toerien M Brookes ST Metcalfe C A review of reporting of participant recruitment and retention in RCTs in six major journals. Trials 2009; 10: 52–52 19591685 10.1186/1745-6215-10-52 PMC2717957

[7] Edwards PJ Roberts I Clarke MJ Methods to increase response to postal and electronic questionnaires. Cochrane Database Syst Rev 2009; (3): MR000008–MR000008 19588449 10.1002/14651858.MR000008.pub4 PMC8941848

[8] Sletto R Pretesting of questionnaires. Am Sociol Rev 1940; 5: 193–200

[9] Rodgers A Corbett T Bramley D Do u smoke after txt? Results of a randomised trial of smoking cessation using mobile phone text messaging. Tob Control 2005; 14: 255–61 16046689 10.1136/tc.2005.011577 PMC1748056

[10] Munoz RF Barrera AZ Delucchi K International Spanish/English Internet smoking cessation trial yields 20% abstinence rates at 1 year. Nicotine Tob Res 2009; 11: 1025–34 19640833 10.1093/ntr/ntp090 PMC2725004

[11] Free CKR Rodgers A Whittaker R Txt2stop: a randomised controlled trial of mobile phone based smoking cessation support. Lancet 2008 10.1136/tc.2008.026146 19318534

[12] Free C Whittaker R Knight R Txt2stop: a pilot randomised controlled trial of mobile phone-based smoking cessation support. Tob Control 2009; 18: 88–91 19318534 10.1136/tc.2008.026146

[13] Hoile ECFC Edwards PJ Felix LM Methods to increase response rates for data collected by telephone. Cochrane Database Syst Rev 2009; 3 10.1002/14651858.MR000008.pub4 PMC8941848 19588449

[14] Altman DG Bland JM Treatment allocation by minimisation. BMJ 2005; 330: 843–843 15817555 10.1136/bmj.330.7495.843 PMC556084

[15] Leong KC Chen WS Leong KW The use of text messaging to improve attendance in primary care: a randomized controlled trial. Fam Pract 2006; 23: 699–705 16916871 10.1093/fampra/cml044

[16] Puccio JA Belzer M Olson J The use of cell phone reminder calls for assisting HIV-infected adolescents and young adults to adhere to highly active antiretroviral therapy: a pilot study. AIDS Patient Care STDS 2006; 20: 438–44 16789857 10.1089/apc.2006.20.438

[17] Doward J Postal union in high court bid to block Royal Mail strike breakers. The Observer . Available at: http://www.guardian.co.uk/uk/2009/nov/01/post-union-high-court-bid (2009, accessed July 2010)

